# Supplementary material for: The Reform of School Catering in Hungary: Anatomy of a Health-Education Attempt
Source: Nutrients. 2019 Mar 27;11(4):716. doi: 10.3390/nu11040716 (PMC6521065; doi:10.3390/nu11040716)
Supplement: Supplementary file 1 [file nutrients-11-00716-s001.pdf]

Supplement

Table S1. Synoptic table of school-feeding policy and regulation based on (EU, 2018). Legend: 1 - there is a recommendation/legislation

0 - no legislation	AT	BE-FL	BE-VA	BG	HR	CY	CZ	DK	EE	FI	FR	DE	GR	HU	IE	IT	LT	LU
**School food policy objectives**																		
**Improve child nutrition**	1	1	1	1	1	1	0	1	1	1	1	1	1	1	1	1	1	1
**Learn healthy habits**	1	1	1	1	1	1	0	1	0	1	1	1	1	1	1	0	1	1
**Reduce/prevent obesity**	1	0	1	1	0	1	0	1	1	1	1	1	1	1	1	0	1	1
**Reduce/prevent malnutrition**	1	0	1	1	0	0	0	0	1	1	1	1	1	1	0	1	1	0
**Tackle health inequalities**	1	0	1	0	0	0	0	0	1	1	0	1	0	0	1	0	0	0
**Improve attainment**	1	0	1	0	0	0	0	0	1	0	1	1	1	1	1	1	0	0
**Support parents and local community**	0	0	1	0	0	0	0	1	0	1	0	0	0	0	0	1	1	0
**Support local agriculture**	1	0	1	0	0	0	0	0	0	0	0	0	0	1	0	0	0	0
**Support local economy**	1	0	1	0	0	0	0	0	0	0	0	0	0	0	0	0	0	0
**Improve school attendance**	0	0	0	0	0	0	0	0	0	0	0	0	0	0	1	1	0	0
**Measures of policy evaluation**																		
**Food provision at school**	1	1	1	1	1	0	0	0	1	1	1	0	1	1	0	1	0	0
**Take up of school meals**	1	0	1	1	0	0	0	0	1	1	0	0	0	1	0	1	0	0
**Nutrition of children**	0	0	0	1	1	0	0	0	0	1	0	0	0	1	0	0	0	0
**Food consumption at school**	0	0	1	1	1	0	0	0	1	1	0	0	0	1	0	0	0	0
**Financial viability of services**	1	0	1	0	0	0	0	0	0	0	0	0	0	1	0	1	0	0
**Engagement of local farmers**	0	0	0	0	0	0	0	0	0	0	0	0	0	1	0	1	0	0
**Support of local economy**	0	0	0	0	0	0	0	0	0	0	0	0	0	0	0	0	0	0
**Reduction in health equality**	0	0	0	0	0	0	0	0	0	0	0	0	0	0	0	0	0	0
**Authorized absence (e.g., sickness)**	0	0	0	0	0	0	0	0	0	0	0	0	0	0	0	0	0	0
**Attainment**	0	0	0	0	0	0	0	0	0	0	0	0	0	0	0	0	0	0
**Food policy standards and guidelines**																		
**Food-based standards of non-lunch**	1	1	1	1	1	1	1	1	0	1	1	1	1	1	0	1	1	1

**Food-based standards for lunch**	1	1	1	1	1	0	1	1	1	1	1	1	0	1	1	1	1	1
**Portion sizes**	1	1	1	1	1	1	1	1	1	1	1	1	1	1	0	1	0	0
**Nutrient-based standards for lunch**	0	0	1	1	1	0	1	1	1	1	0	1	0	1	0	1	1	0
**Catering practices**	1	1	1	1	1	0	1	0	1	1	0	1	1	1	0	0	0	0
**Nutrient-based standards for non-lunch**	0	0	1	1	1	0	1	1	1	0	0	0	1	1	0	1	0	0
**Dining spaces and facilities**	0	1	1	0	0	0	1	1	1	1	0	1	0	1	0	1	0	0
**Staff training**	1	1	1	1	0	0	0	0	0	1	0	0	1	1	0	1	0	1
**Recipes**	1	0	0	1	1	1	0	1	1	1	0	1	0	1	0	0	0	0
**Kitchen facilities/equipment**	0	1	1	0	0	0	0	0	1	0	0	0	1	1	0	0	0	0
**Procurement practices**	0	0	1	0	1	0	1	0	0	1	0	1	1	1	0	0	0	0
**Food arrangement presentation**	1	1	1	0	0	0	0	1	0	1	0	1	1	0	0	1	0	0
**Food recovery/wastage**	0	0	1	0	0	0	0	0	0	0	0	0	1	0	0	0	0	0
**Staffing levels**	0	0	1	0	0	0	0	0	0	1	0	0	1	1	0	0	0	0
**Food contact materials**	0	0	0	1	0	0	0	0	0	0	0	0	1	1	0	0	0	0
**Food based standards**																		
**Drinks limited to specific types (lunch)**	1	1	1	1	1	0	0	1	0	1	1	1	0	1	1	0	1	1
**Drinks limited to specific types (other than lunchtime)**	1	1	1	1	1	0	0	1	0	1	0	1	1	1	1	0	1	1
**Fresh drinking water (lunch)**	0	1	1	1	1	0	0	1	1	1	1	1	0	1	1	1	0	0
**Fresh drinking water (other than lunchtime)**	0	1	1	1	1	0	0	1	0	1	1	1	0	1	1	0	0	0
**Fruit and vegetable provision (lunch)**	1	1	1	1	1	0	1	1	1	1	1	1	0	1	1	1	1	0
**Fruit and vegetable provision (other than lunchtime)**	1	1	1	1	1	1	1	1	0	1	1	1	0	1	1	1	0	0
**Soft drinks now allowed (lunch)**	1	1	1	0	1	0	0	1	1	1	0	1	0	1	1	0	0	1
**Soft drinks now allowed (other than lunchtime)**	0	0	1	1	1	0	0	1	0	1	0	1	1	1	1	0	1	1
**Sweet treats restricted (lunch)**	1	1	1	1	1	0	0	1	1	1	1	1	0	1	1	0	1	0

**Sweet treats restricted (other than lunchtime)**	1	1	1	1	1	1	0	1	0	1	1	1	1	1	1	0	1	0
**Salt provision restricted (lunch)**	1	0	1	1	1	0	0	0	0	1	1	1	0	1	0	1	1	0
**Salt provision restricted (other than lunchtime)**	1	0	0	1	1	1	0	0	0	1	1	1	1	1	0	0	1	0
**Deep fried processed products restricted (lunch)**	1	1	1	1	1	0	0	1	1	0	1	1	0	1	0	0	1	0
**Deep fried processed products restricted (other than lunchtime)**	1	1	1	1	1	1	0	1	0	0	1	1	0	1	0	0	0	1
**Frequency of serving dairy (lunch)**	1	0	1	1	1	0	1	1	0	1	1	1	0	1	1	1	1	1
**Crisps/savory snacks restricted (lunch)**	1	0	1	1	1	0	0	1	0	1	0	1	0	1	1	0	0	0
**Crisps/savory snacks restricted (other than lunchtime)**	1	0	1	1	1	1	0	1	0	1	0	1	0	1	1	0	1	0
**Frequency of serving oily fish (lunch)**	0	1	1	1	1	0	1	1	0	0	0	1	0	0	0	1	1	0
**Frequency of serving non-meat/non-dairy protein (lunch)**	0	1	1	1	1	0	1	0	1	0	0	1	0	0	1	1	0	0
**Starchy food cooked in fat/oil restricted (lunch)**	1	0	1	1	1	0	0	1	1	0	0	1	0	1	0	0	1	1
**Starchy food cooked in fat/oil restricted (other than lunchtime)**	1	0	1	1	1	0	0	1	0	0	0	1	0	1	0	0	0	1
**Frequency of serving (red) meat (lunch)**	1	1	1	0	0	0	1	0	0	0	1	1	0	1	0	0	1	0
**Nutrients-based standards**																		
**Energy (lunch)**	0	0	1	1	1	0	1	1	1	1	0	1	0	1	0	1	1	0
**Energy (other than lunchtime)**	0	0	1	1	1	0	1	1	1	0	0	0	0	1	0	0	0	0
**Fat (lunch)**	0	0	1	1	1	0	1	1	1	1	0	1	0	1	0	1	1	0
**Fat (other than lunchtime)**	0	0	1	1	1	0	1	0	1	1	0	0	1	1	0	0	0	0
**Protein (lunch)**	0	0	1	1	1	0	0	1	1	1	0	1	0	0	0	1	1	0
**Protein (other than lunchtime)**	0	0	1	1	1	0	0	0	1	0	0	0	0	0	0	0	0	0
**Total CHO (lunch)**	0	0	1	1	1	0	0	1	1	1	0	1	0	0	0	1	1	0

**Total CHO (other than lunchtime)**	0	0	1	1	1	0	0	1	1	1	0	0	0	0	0	0	0	0
**Fibre (lunch)**	0	0	1	1	1	0	0	0	1	1	0	1	0	0	0	1	0	0
**Fibre (other than lunchtime)**	0	0	1	1	1	1	0	1	1	0	0	0	0	0	0	0	0	0
**Vitamin C (lunch)**	0	0	1	1	1	0	1	1	1	1	0	1	0	0	0	0	0	0
**Vitamin C (other than lunchtime)**	0	0	1	1	1	0	1	0	1	0	0	0	0	0	0	0	0	0
**Calcium (lunch)**	0	0	1	1	1	0	0	0	1	1	0	1	0	1	0	1	0	0
**Calcium (other than lunchtime)**	0	0	1	1	1	0	0	0	1	0	0	0	0	1	0	0	0	0
**Iron (lunch)**	0	0	1	1	1	0	0	0	1	1	0	1	0	0	0	1	0	0
**Iron (other than lunchtime)**	0	0	1	1	1	0	0	0	1	0	0	0	0	0	0	0	0	0
**Sodium (lunch)**	0	0	1	1	1	0	0	0	1	1	0	1	0	1	0	0	1	0
**Sodium (other than lunchtime)**	0	0	1	1	1	0	0	0	1	0	0	0	1	1	0	0	0	0
**Saturated fat (lunch)**	0	0	1	1	1	0	0	1	1	1	0	1	0	0	0	1	0	0
**Saturated fat (other than lunchtime)**	0	0	1	1	1	0	0	1	1	0	0	0	1	0	0	0	0	0
**Folate (lunch)**	0	0	1	1	1	0	0	0	1	0	0	1	0	0	0	0	0	0
**Folate (other than lunchtime)**	0	0	1	1	1	0	0	0	1	0	0	0	0	0	0	0	0	0
**Sugars (lunch)**	0	0	1	1	1	0	1	0	1	0	0	1	0	1	0	0	1	0
**Sugars (other than lunchtime)**	0	0	1	1	1	1	1	0	1	0	0	0	1	1	0	0	0	0
**Vitamin A (lunch)**	0	0	1	1	1	0	0	0	1	0	0	1	0	0	0	0	0	0
**Vitamin A (other than lunchtime)**	0	0	1	1	1	0	0	0	1	0	0	0	0	0	0	0	0	0
**Zinc (lunch)**	0	0	1	1	1	0	0	0	1	0	0	1	0	0	0	0	0	0
**Zinc (other than lunchtime)**	0	0	1	1	1	0	0	0	1	0	0	0	0	0	0	0	0	0

	MT	NL	NO	PL	PT	RO	SK	SL	ES	SE	CH	GB-ENG	GB-NIR	GB-SCT	GB-WLS
**School food policy objectives**															
**Improve child nutrition**	1	1	1	1	1	1	1	1	1	1	1	1	1	1	1
**Learn healthy habits**	1	1	1	1	1	1	1	1	1	1	1	1	1	1	1
**Reduce/prevent obesity**	1	1	0	1	1	1	1	1	1	1	1	1	1	1	1
**Reduce/prevent malnutrition**	1	1	0	1	1	1	1	1	1	1	1	1	0	1	0
**Tackle health inequalities**	0	0	1	1	1	0	1	1	1	1	1	1	1	1	1

**Improve attainment**	0	1	1	0	1	0	1	1	0	1	0	1	1	1	1
**Support parents and local community**	1	0	0	0	0	0	1	1	1	0	0	1	0	1	1
**Support local agriculture**	0	0	0	0	0	1	0	1	0	0	1	0	0	1	0
**Support local economy**	0	0	0	0	0	0	0	1	0	0	0	0	0	1	0
**Improve school attendance**	0	1	0	0	0	0	0	0	0	0	0	1	0	1	1
**Measures of policy evaluation**															
**Food provision at school**	1	1	1	0	1	0	0	1	1	1	0	0	1	1	1
**Take up of school meals**	0	0	0	0	0	0	0	1	1	0	0	1	1	1	1
**Nutrition of children**	0	0	0	1	0	1	0	1	0	0	0	0	1	1	1
**Food consumption at school**	0	0	0	0	0	0	0	1	1	0	0	0	1	1	0
**Financial viability of services**	0	0	0	0	0	0	0	1	0	0	0	0	0	1	0
**Engagement of local farmers**	0	0	0	0	0	0	0	1	0	0	0	0	0	1	0
**Support of local economy**	0	0	0	0	0	0	0	0	0	0	0	0	0	1	0
**Reduction in health equality**	0	0	0	0	0	0	0	1	0	0	0	0	0	1	0
**authorized absence (e.g., sickness)**	0	0	0	0	0	0	0	1	0	0	0	0	0	0	0
**Attainment**	0	1	0	0	0	0	0	0	0	0	0	0	0	0	0
**Food-based standards of non-lunch**	1	1	1	0	1	1	1	1	1	1	1	0	1	1	1
**Food policy standards and guidelines**															
**Food-based standards for lunch**	1	1	1	0	1	0	1	1	1	1	1	1	1	1	1
**Portion sizes**	1	0	0	0	1	1	1	1	1	0	1	1	1	1	1
**Nutrients based standards for lunch**	1	1	0	1	0	0	1	1	1	1	1	0	0	1	1
**Catering practices**	0	1	0	0	0	1	1	0	1	0	1	1	1	0	0
**Nutrient-based standards for non-lunch**	1	1	0	1	0	1	1	1	1	0	1	0	0	0	0
**Dining spaces and facilities**	0	0	1	0	0	1	1	1	1	1	1	1	1	1	1
**Staff training**	0	0	0	1	0	1	1	1	1	0	1	0	1	0	1
**Recipes**	1	0	0	1	0	0	1	1	1	0	1	0	1	0	0
**Kitchen facilities/equipment**	1	0	0	1	0	1	1	1	0	0	1	0	1	0	0
**Procurement practices**	0	0	0	0	0	1	0	1	0	0	1	0	1	1	0

**Food arrangement presentation**	0	0	0	0	0	1	0	1	1	1	1	0	1	0	0
**Food recovery/wastage**	0	0	0	0	0	1	1	1	1	1	1	0	1	0	0
**Staffing levels**	0	0	0	0	0	1	1	1	1	0	0	0	0	0	0
**Food contact materials**	0	0	0	0	0	1	1	1	1	0	0	0	0	0	0
**Food based standards**															
**Drinks limited to specific types (lunch)**	1	1	1	0	1	1	1	1	1	1	1	1	1	1	1
**Drinks limited to specific types (other than lunchtime)**	0	1	1	0	1	1	1	1	1	1	1	1	1	1	1
**Fresh drinking water (lunch)**	1	1	1	0	1	1	1	1	1	1	1	1	1	1	1
**Fresh drinking water (other than lunchtime)**	0	1	1	0	0	1	1	1	1	1	1	0	1	1	1
**Fruit and vegetable provision (lunch)**	0	0	1	0	1	0	1	1	1	1	1	1	1	1	1
**Fruit and vegetable provision (other than lunchtime)**	0	0	1	0	0	1	1	1	1	1	1	1	1	1	1
**Soft drinks now allowed (lunch)**	1	0	1	0	1	1	0	1	1	1	1	1	1	1	1
**Soft drinks now allowed (other than lunchtime)**	0	0	1	0	1	1	0	1	1	1	1	1	1	1	1
**Sweet treats restricted (lunch)**	0	0	1	0	0	0	0	1	1	1	1	0	1	1	1
**Sweet treats restricted (other than lunchtime)**	0	0	1	0	1	1	1	1	1	1	1	1	1	1	1
**Salt provision restricted (lunch)**	1	0	0	0	1	1	0	1	1	1	1	0	1	1	1
**Salt provision restricted (other than lunchtime)**	0	0	0	0	0	1	0	1	1	0	1	1	1	1	1
**Deep fried processed products restricted (lunch)**	0	1	0	0	1	0	0	1	1	0	1	1	1	1	1
**Deep fried processed products restricted (other than lunchtime)**	0	1	0	0	1	1	0	1	0	0	1	1	1	1	1
**Frequency of serving dairy (lunch)**	0	0	0	1	0	0	0	1	1	1	1	1	1	1	1
**Crisps/savory snacks restricted (lunch)**	1	0	1	0	0	0	0	1	1	1	1	1	1	1	1

**Crisps/savory snacks restricted (other than lunchtime)**	1	0	1	0	1	1	0	1	1	1	1	1	1	1	1
**Frequency of serving oily fish (lunch)**	0	0	0	0	0	0	1	1	1	1	1	1	1	1	1
**Frequency of serving non-meat/non-dairy protein (lunch)**	0	0	0	0	0	0	0	1	1	1	1	1	1	1	1
**Starchy food cooked in fat/oil restricted (lunch)**	0	0	0	0	0	0	0	1	1	0	1	1	1	1	1
**Starchy food cooked in fat/oil restricted (other than lunchtime)**	0	0	0	0	1	1	0	1	0	1	1	1	1	1	1
**Frequency of serving (red) meat (lunch)**	0	1	0	0	0	0	1	1	1	1	0	1	1	1	1
**Nutrients-based standards**															
**Energy (lunch)**	0	1	0	1	0	0	1	1	1	1	1	1	0	1	1
**Energy (other than lunchtime)**	0	0	0	0	0	1	1	1	1	1	1	0	0	0	0
**Fat (lunch)**	0	0	0	1	0	0	1	1	0	1	1	1	0	1	1
**Fat (other than lunchtime)**	0	0	0	0	0	1	1	1	1	0	1	0	0	0	0
**Protein (lunch)**	0	0	0	1	0	1	1	1	0	1	1	1	0	1	1
**Protein (other than lunchtime)**	0	0	0	0	0	0	1	1	0	0	1	0	0	0	0
**Total CHO (lunch)**	0	0	0	1	0	0	0	1	0	1	1	1	0	1	1
**Total CHO (other than lunchtime)**	0	0	0	0	0	0	0	1	0	0	1	0	0	0	0
**Fibre (lunch)**	0	0	0	1	0	0	1	1	0	1	1	1	0	1	1
**Fibre (other than lunchtime)**	0	0	0	0	0	0	0	1	0	0	1	0	0	0	0
**Vitamin C (lunch)**	0	0	0	1	0	0	1	1	0	1	1	1	0	1	1
**Vitamin C (other than lunchtime)**	0	0	0	0	0	0	1	1	0	0	1	0	0	0	0
**Calcium (lunch)**	0	0	0	1	0	0	1	1	0	0	1	1	0	1	1
**Calcium (other than lunchtime)**	0	0	0	0	0	0	0	1	0	0	1	0	0	0	0
**Iron (lunch)**	0	0	0	1	0	0	1	1	0	1	1	1	0	1	1
**Iron (other than lunchtime)**	0	0	0	0	0	0	0	1	0	0	1	0	0	0	0
**Sodium (lunch)**	0	0	0	1	0	0	1	1	0	1	1	1	0	1	1
**Sodium (other than lunchtime)**	0	0	0	0	0	1	0	1	1	0	1	0	0	0	0

**Saturated fat (lunch)**	0	0	0	1	0	0	0	1	0	1	0	1	0	1	1
**Saturated fat (other than lunchtime)**	0	0	0	0	0	1	0	1	1	0	0	0	0	0	0
**Folate (lunch)**	0	0	0	1	0	0	1	1	0	1	1	1	0	1	1
**Folate (other than lunchtime)**	0	0	0	0	0	0	0	1	0	0	1	0	0	0	0
**Sugars (lunch)**	0	0	0	1	0	0	1	1	0	0	0	1	0	1	1
**Sugars (other than lunchtime)**	0	0	0	0	0	0	1	1	1	0	0	0	0	0	0
**Vitamin A (lunch)**	0	0	0	1	0	0	1	1	0	0	0	1	0	1	1
**Vitamin A (other than lunchtime)**	0	0	0	0	0	0	0	1	0	0	0	0	0	0	0
**Zinc (lunch)**	0	0	0	1	0	0	1	1	0	0	0	1	0	1	1
**Zinc (other than lunchtime)**	0	0	0	0	0	0	0	1	0	0	0	0	0	0	0

Source: Authors' own construction based on EU [28]

Table S2. Structure of participants in preliminary phase of research.

	Preliminary interview phase	
	**no. of participants**	**%**
**Catering service entrepreneur**	2	8.33
**Catering service manager (dietician)**	3	12.50
**Teachers**	5	20.83
**Independent nutritional specialists**	4	16.67
**Government**	1	4.17
**Local authority**	2	8.33
**Parents**	7	29.17
**Total**	24	100

Source: Authors' own construction

Table S3. The socio-economic characteristic features of participants (n = 72).

	**Highest level of qualification**			**Gender**		**Age**			**Place of residence**			
	**High school**	**BSC**	**MSc or higher**	**male**	**female**	**<35**	**36–50**	**>50**	**Budapest (capital)**	**Big town**	**Small town**	**Village**
**Catering service entrepreneur (n = 6)**	1	3	2	3	3	1	4	1	2	1	1	2
**Catering service manager (n = 12)**	2	8	2	1	11	2	8	2	6	1	3	2
**Independent nutritional specialists (n = 6)**			6		6	3	2	1	6			
**Government (n = 5)**			5	2	3	1	2	2	5			
**Local authority (n = 4)**		1	3	2	2		1	3	1	1	1	1
**Parents (n = 26)**	13	9	4	5	21	13	12	1	12	4	5	5
**Teachers (n = 13)**		9	4	1	12	2	7	4	3	4	3	3

Source: Authors' own construction
